# Phase I/IIa study of intratumoral/intracerebral or intravenous/intracerebral administration of Parvovirus H-1 (ParvOryx) in patients with progressive primary or recurrent glioblastoma multiforme: ParvOryx01 protocol

**DOI:** 10.1186/1471-2407-12-99

**Published:** 2012-03-21

**Authors:** Karsten Geletneky, Johannes Huesing, Jean Rommelaere, Joerg R Schlehofer, Barbara Leuchs, Michael Dahm, Ottheinz Krebs, Magnus von Knebel Doeberitz, Bernard Huber, Jacek Hajda

**Affiliations:** 1 Department of Neurosurgery, University Hospital Heidelberg, Im Neuenheimer Feld 400, Heidelberg 69120, Germany; 2 Coordination Centre for Clinical Trials, University Hospital Heidelberg, Vossstr. 2, Building 4410, Heidelberg 69115, Germany; 3 Department of Applied Tumor Virology, German Cancer Research Center, Im Neuenheimer Feld 242, Heidelberg 69120, Germany; 4 Oryx GmbH & Co KG, Marktplatz 1, Baldham 85598, Germany; 5 Department of Applied Tumor Biology, Institute of Pathology, University Hospital Heidelberg, Im Neuenheimer Feld 220/221, Heidelberg 69120, Germany

## Abstract

**Background:**

The treatment of patients with malignant brain tumors remains a major oncological problem. The median survival of patients with glioblastoma multiforme (GBM), the most malignant type, is only 15 months after initial diagnosis and even less after tumor recurrence. Improvements of standard treatment including surgery and radio-chemotherapy have not lead to major improvements. Therefore, alternative therapeutics such as oncolytic viruses that specifically target and destroy cancer cells are under investigation. Preclinical data of oncolytic parvovirus H-1 (H-1PV) infection of glioma cells demonstrated strong cytotoxic and oncosuppressing effects, leading to a phase I/IIa trial of H-1PV in patients with recurrent GBM (ParvOryx01). ParvOryx01 is the first trial with a replication competent oncolytic virus in Germany.

**Methods:**

ParvOryx01 is an open, non-controlled, two groups, intra-group dose escalation, single center, phase I/IIa trial. 18 patients with recurrent GBM will be treated in 2 groups of 9 patients each. Treatment group 1 will first receive H-1PV by intratumoral injection and second by administration into the walls of the tumor cavity during tumor resection. In treatment group 2 the virus will initially be injected intravenously and afterwards, identical to group 1, into the surrounding brain tissue during tumor removal. Main eligibility criteria are: age of 18 years, unifocal recurrent GBM, amenable to complete or subtotal resection. Dose escalation will be based on the Continual Reassessment Method. The primary objective of the trial is local and systemic safety and tolerability and to determine the maximum tolerated dose (MTD). Secondary objectives are proof of concept (PoC) and Progression-free Survival (PFS) up to 6 months.

**Discussion:**

This is the first trial with H-1PV in patients with recurrent GBM. The risks for the participants appear well predictable and justified. Furthermore, ParvOryx01 will be the first assessment of combined intratumoral and intravenous application of an oncolytic virus. Due to its study design the trial will not only generate data on the local effect of H-1PV but it will also investigate the penetration of H-1PV into the tumor after systemic delivery and obtain safety data from systemic delivery possibly supporting clinical trials with H-1PV in other, non-CNS malignancies.

**Trial registration:**

ClinicalTrials.gov Identifier: NCT01301430

## Background

According to the estimation of the American Cancer Society about 16,000 new cases of intracranial neoplasm are diagnosed in the USA each year, being over half the number of new cases of melanoma [1]. The largest group with approximately 12,700 new cases per year are gliomas, primary central nervous system neoplasms with characteristics of neuroglial cells. Of those the most frequent subgroup with more than 6,000 new diagnoses per year is glioblastoma multiforme (GBM), which is also the most malignant type of gliomas. The incidence of GBM is currently showing a slight but relevant increase [2]. The majority of patients diagnosed with GBM is over 40 years of age [1].

Despite different therapeutic approaches and their recent progress, the treatment options for patients suffering from glioblastoma are still disappointing. The overall survival rates amounted to 33, 12, 7, 5, and 4 per cent after 1, 2, 3, 4, and 5 years, respectively [3]. The 1-year PFS was as low as 10% in individuals presenting recurrent disease and treated solely with chemotherapy [4]. At present, established therapeutic strategies include surgery, radiation, chemotherapy, and molecular targeted agents. New approaches are on the way [5].

Amongst other experimental therapies, oncolytic viruses are investigated for treatment of high-grade gliomas. Several candidates such as HSV-1, adenovirus, Newcastle disease virus, reovirus, poliovirus, and vaccinia virus were identified to have toxic effects on glioma cells [6]. Up to now several clinical trials were carried out to demonstrate the safety and in part efficacy of the viral candidates in patients suffering from recurrent malignant gliomas [7-11]. All viruses were administered either intratumorally or intracerebrally into the walls of the resection cavity. A good tolerability and no safety issues could be observed throughout the trials. If applicable, the dose escalation could be performed according to the predefined schedule.

## Trial rationale/justification

Agents that possess anti-glioma activity, a tolerable safety profile and no unjustified risk for the general population should undergo rapid development to assess their therapeutic potential.

Parvovirus H-1 (H -1PV) is a small single-stranded rodent DNA virus. The natural host is rat but H-1PV is able to infect and replicate in cells of various other species including humans. Parvoviruses are unique among DNA viruses as they do not have any tumorigenic members [12]. In contrast, some parvoviruses exert cytopathic effects, mainly in neoplastic cells [13] and they have been shown to possess oncosuppressive properties [14,15]. In general, parvovirus H-1 is classified as non pathogenic.

A strong oncolytic effect was shown in different established glioblastoma cell lines of rat and human origin and in short-term/low-passage cultures of human glioblastoma cells, at low multiplicities of infection (MOI) [16]. Infection of glioma cells with H-1PV virus yielded progeny H-1PV virions in all cultures, however with marked differences of titers. In conclusion, malignant glial cells appear to represent a highly susceptible target for H-1PV virus-mediated cytotoxicity. Furthermore, H-1PV was shown to activate a non-conventional, cathepsin-mediated death pathway, leading to cell killing also in glioma cells that are frequently resistant to apoptotic cell death mechanisms [17].

In orthotopic glioma models in rats large tumors (> 6 mm) were either injected stereotactically with H-1PV (single intratumoral injection) or H-1PV was administered by multiple intravenous injections (systemic route). H-1PV virus treatment resulted in rapid tumor regression and significant prolongation of survival of treated animals [18]. Histological analysis showed widespread destruction of tumor tissue, without any toxic or inflammatory side effects in the surrounding brain tissue. Virus replication was demonstrated in tumors, indicating a contribution of secondary infection by progeny virus to the efficiency of oncolysis. Hence, complete remission of advanced intracranial gliomas by oncolytic parvovirus H-1PV could be achieved showing that H-1PV possesses strong anti-tumor activity also in vivo.

Based on the findings described above, H-1PV may be reasonably assumed to be effective for treatment of glioblastoma in humans. Moreover, the virus offers some potential advantages over the current therapeutic modalities:

Unlike other oncolytic viruses, H-1PV was shown to cross the blood-brain barrier and to infect intracerebral tumors. This offers the chance of boosting the initial local therapy by consecutive intravenous administrations or for interval retreatment without the necessity of craniotomy.

H-1PV may potentially evoke an anticancer vaccination effect based on release of tumor-associated antigens and subsequent immunostimulation. This could lead to long-term effects in prevention of disease relapse, potentially adding to initial oncolysis.

Taken together, the therapy with H-1PV as planned in this protocol may provide considerable benefit for included subjects. Furthermore, the findings from the trial are crucial for consecutive clinical development of H-1PV for glioblastoma and other tumor entities.

## Design and methods

### Aim

This trial aims to investigate the safety, biodistribution, maximum tolerated dose and signs of anti-tumor activity of parvovirus H-1 in patients suffering from recurrent malignant gliomas. According to preclinical data and differing from previous oncolytic virus trials in GBM patients ParvOryx01 will not only include intratumoral virus application but also intravenous treatment.

### Objectives

#### Primary objectives

The primary objective of the trial is related to the safety and tolerability of the Investigational Medicinal Product (IMP). The particular parameters for this objective are:

• Local and systemic safety and tolerability of intratumoral/intracerebral and intravenous/intracerebral administration of the IMP,

• Maximum tolerated dose (MTD) of the IMP as assessed by NCI CTCAE v4.0,

• Viremia following intratumoral, intracerebral and intravenous administration of the IMP,

• IMP shedding/persistence following intratumoral, intracerebral and intravenous administration of the IMP.

#### Secondary objectives

The secondary objective of the study is related to the efficacy of the IMP. The particular parameters for this objective are:

• Evidence for anti-tumor activity of the IMP in subjects with recurrent GBM (Proof of Concept, PoC),

• Progression-free Survival (PFS) up to 6 months after study inclusion,

• Overall survival (OS) up to 6 months after study inclusion.

### Design

Parvoryx01 is an open, non-controlled, two groups, intra-group dose escalation, single center study.

Due to the exploratory approach of the current trial with regard to safety and tolerability of the IMP no positive control will be used. The implementation of a negative control (placebo) is ethically unjustifiable in subjects suffering from rapid progressive disease who otherwise can undergo a potentially effective treatment.

The safety and tolerability of the IMP will be investigated after both, local, i.e. intratumoral/intracerebral and systemic, i.e. intravenous administration. Therefore, two separate study groups representing both modes of administration will be examined. Since the trial is the first-in-man study of the IMP, the dose escalation will be carried out in accordance to a sequential escalating design, including interims assessments of safety and tolerability after each dose level and between both study groups.

Due to the complex handling and administration of the IMP this first trial will be performed in a single center under coordination of an investigator who possesses profound knowledge of all aspects concerning the IMP.

### Eligibility

Patients have to suffer from progressive primary or recurrent glioblastoma multiforme (GBM) scheduled for neurosurgery, i.e. for a complete or subtotal tumor resection. Patients have to fulfill the following main inclusion crieteria: (i) 18 years of age or older, (ii) confirmed diagnosis of glioblastoma multiforme (WHO grade IV), (iii) recurrence or tumor progression despite previous radio- and/or chemotherapy, (iv) life expectancy of at least 3 months, (v) Karnofsky Performance Score ≥60, (vi) commitment to omit exposure to infants < 18 months of age or immunocompromised individuals for up to 28 day after first administration of IMP.

Main criteria to exclude subjects from the trial are (i) multifocal disease, (ii) evidence of distant tumor metastases, (iii) contraindications for MRI, (iv) treatment with antiangiogenic substances within 21 days prior to therapy, (v) radiotherapy within 90 days prior to study inclusion, (vi) chemotherapy within 4 weeks prior to study inclusion.

### Sample size

The current trial represents the first in man study with the IMP (ParvOryx). 18 subjects will be allocated to 2 study groups with 9 subjects per group. Per group, 3 dose levels will be investigated. The mean number of 3 subjects per dose level appears to be sufficient to give first indications about safety, tolerability and proof of concept of ParvOryx.

There will be no a-priori defined quantitative ratio between females and males. However, no gender differences in the safety, tolerability as well as efficacy are expected on the basis of preclinical findings.

### Course of the trial

For each patient the ParvOryx01- trial includes two phases: i) the screening phase in which eligibility for trial inclusion is assessed and ii) the treatment/follow-up phase in which the trial treatment, the medical monitoring and 3 follow-up visits are performed. Due to the rapid growth of recurrent glioblastomas and the limited capacity for fast consecutive study inclusion according to safety regulations, patients have to be continuously screened while other patients are already under treatment. Therefore, screening phase and treatment phase are overlapping.

#### Screening phase

Screening of patients has to be performed within 2 weeks prior to study inclusion. Potentially eligible patients are provided with comprehensive written and verbal information. Investigations that are carried out during the screening procedure include:

Written informed consent, demography and medical history, physical and neurological examination, vital signs and 12-lead ECG, clinical chemistry, hematology and clotting, serum protein electrophoresis, MRI, viral shedding of H-1 PV in blood, urine, stool and throat swabs, H-1 PV specific antibodies, HIV, HBV and HCV serology, pregnancy test.

At discretion of a responsible investigator patients in group II can be required to undergo a stereotactic biopsy and sampling of a tumor specimen for histological confirmation of GBM by a neuropathologist (only, if in opinion of the investigator the evidence of glioblastoma recurrence is not sufficient).

#### Intervention

**Figure 1:**
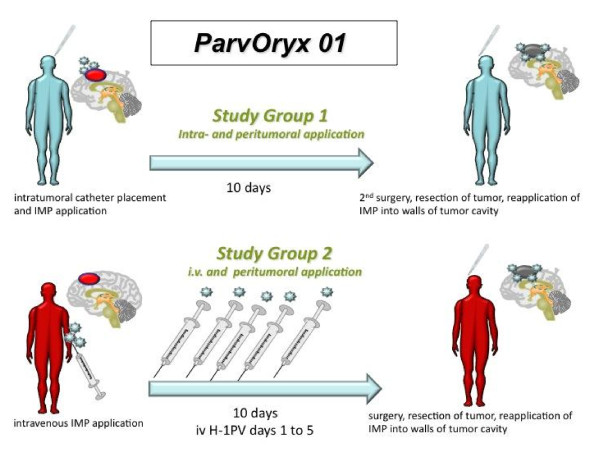
**Schematic diagram of the ParvOryx01 trial: study group 1 will be treated first**. After completion of group 1 an interim analysis will be performed prior to recruiting patients for study group 2. Information about dose escalation is specified in Table 1.

The IMP for this trial is ParvOryx, a GMP-grade preparation of Parvovirus H-1. The application of the IMP will be performed in 2 groups of 9 patients each. The route of administration differs between group 1 and group 2 (Figure 1). Within each group the mode of application is identical, but the dose will be increased if no dose limiting events are observed. In each group the IMP will be administered in three dose levels (Table 1)

**Table 1:** Dose schedule for both study groups

GROUP I			
Escalation Level	Study Time	Dose and route of administration	Duration
**Level 1** Total dose: 1 × 10^6 ^pfu	Day 1	5 × 10^5 ^pfu, intratumoral (via catheter)	15 minutes
	
	Day 10	5 × 10^5 ^pfu, intracerebal (direct injection at multiple locations of resection wall)	15-30 minutes

**Level 2** Total dose: 5 × 10^7 ^pfu	Day 1	2.5 × 10^7 ^pfu, intratumoral (via catheter)	15 minutes
	
	Day 10	2.5 × 10^7 ^pfu, intracerebal (direct injection at multiple locations of resection wall)	15-30 minutes

**Level 3** Total dose: 1 × 10^9 ^pfu	Day 1	5 × 10^8 ^pfu, intratumoral (via catheter)	15 minutes
	
	Day 10	5 × 10^8 ^pfu, intracerebral (direct injection at multiple locations of resection wall)	15-30 minutes

GROUP II			
**Escalation Level**	**Study Time**	**Dose and Route of Administration**	**Duration**

**Level 1 **Total dose: 1 × 10^6 ^pfu	Day 1 - 5	1 × 10^5 ^pfu, intravenous infusion	2 hours
	
	Day 10	5 × 10^5 ^pfu, intracerebral (direct injection at multiple locations of resection wall)	15-30 minutes

**Level 2** Total dose: 5 × 10^7^	Day 1 - 5	0.5 × 10^7 ^pfu, intravenous infusion	2 hours
	
	Day 10	2.5 × 10^7 ^pfu, intracerebral (direct injection at multiple locations of resection wall)	15-30 minutes

**Level 3 **Total dose: 1 × 10^9 ^pfu	Day 1 - 5	1 × 10^8 ^pfu, intravenous infusion	2 hours
	
	Day 10	5 × 10^8 ^pfu, intracerebral (direct injection at multiple locations of resection wall)	15-30 minutes

Specification of virus application and dose escalation during the Parvoryx01 trial: dose group 2 will be treated after completion of group 1 and after interim analysis of safety and tolerability in group 1.

In group 1 the patients will receive the IMP on day 1 via image guided injection into the tumor tissue. On this day the patient is injected with 50% of the intended overall dose. After an observation period of 9 days the tumor will be resected on day 10. After tumor removal the second half of the dose will be administered into the walls of the resection cavity by direct injection. With this injection during open surgery the administration of the IMP is completed and no additional virus application will be performed.

In group 2 the initial administration of the IMP is via the intravenous route. Subjects receive 50% of the intended dose by 5 infusions on days 1 to 5, each infusion containing 10% of the total dose. After the last infusion on day 5 there is an observation period until day 9 and on day 10 tumor resection will be performed as in group 1. In analogy to group 1, patients receive the second half of the dose by injection in the tissue surrounding the tumor cavity after tumor removal and no further virus injections will be performed in each individual during the course of the trial.

#### Trial schedule and duration

As ParvOryx01 is a first in man trial with administration of a replication competent parvovirus the recruitement schedule for the trial is strictly regulated. Prior to the treatment of the 2nd subject of each dose level data from the 1st subject of this level will be collected up to day 28. Similarly, prior to the treatment of the 3rd subject of a dose level data from the 2nd subject up to day 18 will be collected. The 2nd and 3rd subject will only undergo treatment with the IMP if no unjustifiable adverse reactions occurred previously. The decision will be made by mutual agreement between the principal investigator or another responsible investigator and the sponsor. After completion of a dose level the data safety monitoring board (DSMB) has to give consent to move to the next dose level. A decision regarding the progress to the 2nd study group will be based on agreement between DSMB, principal investigator and sponsor after review of selected data from 1st study group obtained up to Day 28. The information obtained from 1st study group will be provided to the Competent Authority (Paul-Ehrlich-Institute) for a review. A progress to the 2nd study group will only take place if there is no objection of the Competent Authority.

The per-subject trial duration including all follow-up visits will be 6 months. After the end of the main treatment phase on day 28, 3 follow-up visits will be performed at 2, 4 and 6 months after trial inclusion. The overall duration of the clinical trial, including completion of all follow-up visits related to the efficacy of the IMP in all subjects, is scheduled to last approximately 24 months.

The treatment phase and the capture of data on the safety and tolerability of the IMP (primary objective) will be completed approximately 18 month after inclusion of the 1st study subject. The per-subject trial duration as related to the primary objective will last 28 days.

It can be assumed that the treatment and the documentation of the primary objective parameters in one dose escalation level will take approximately 3 months.

### Specific hygienic measures

Due to the first in man application of a replication competent parvovirus and the lack of data about the toxicity and excretion of the IMP in humans after intracerebral or intravenous injection specific procedures have to be applied. Despite low hygienic risks associated with the current trial a number of hygienic measures have to be adhered to (Table 2).

**Table 2:** Specific hygenic measures

• Information of staff involved in the medical care/nursing of trial subjects (e.g. physicians, nurses, cleaning staff, physiotherapist),
• Proposal of serological testing of medical staff for H-1PV prior to the beginning and at the end of the trial (in order to document potential seroconversion),
• Consideration of a subject as potentially viremic until:
○ A seroconversion as proven by HIT or ELISA, ○ In the absence of a seroconversion until proven lack of virus shedding in urine, saliva, and feces,
• While a subject is considered as potentially viremic:
○ Isolate her/him in a single room with its own toilet, ○ Use disposable gloves, protection gown and mask for handling of study medication and materials contaminated with ParvOryx, e.g. infusion materials, ○ Information of medical staff to use gloves, protection gown and mask when in physical contact with the patient, ○ Information of medical staff and visitors to use mask only when strictly omitting body contact with the subject during his/her isolation phase. Alternatively, the subject can wear a mask when in the same room with medical staff and visitors, ○ Information of hygiene staff to carry out a terminal disinfection after discharge of a subject from isolation, ○ In case of surface contamination the disinfection must be performed with Perform^®^, 3%

ParvOryx01 is the first in man application of a GMP grade oncolytic parvovirus. The hygienic precautions listed above must be applied including isolation of patients in a single room as long as they are considered viremic. Virus shedding and seroconversion are tested daily to reduce duration of isolation to a minimum

### Benefit/risk assessment

Currently, recurrent glioblastoma is a tumor entity for which no established standard of care exists. The prognosis of this disease is dismal, the progression free survival at 6 months (6 M-PFS) ranges between 15% to 21% and the median survival of patients is in the range of 25 weeks [19]. Re-operation is one possible treatment option with proven clinical benefit. It can be estimated that one third of patients with recurrent GBM are candidates for a second debulking surgical procedure.

Non-surgical treatment options include the application of re-irradiation for a subset of approximately 20% of patients and the use of chemotherapy. None of these treatments has been shown to have a major impact on PFS or survival.

The use of replication competent Parvovirus H-1 in the current trial setting therefore addresses a patient population without a standard of care and with only limited treatment options. According to the study protocol even without oncolytic efficacy of H-1PV patients would benefit from participating in the trial due to re-operation after initial virus treatment.

There is only limited information about the effects of Parvovirus H-1 infections of humans, but according to the assessment of the pathogenicity of the virus in humans by the Robert Koch Institut it is classified as non-pathogenic in man. This is supported by the limited available information from human patients. According to data from Parvovirus H-1 application in terminally ill tumor patients in 1965 and in 1993 the injection(s) did not cause severe side effects. The virus that was used in these patients (n = 2 in 1965 and n = 12 in 1993) was not produced according to today's GMP standard and the amount of virus that was injected was in the range from approx. 2 × 10 exp8 to approx. 3 × 10 exp10 [20,21]. Recently, it could also be demonstrated that even laboratory staff who had worked with Parvovirus H-1 for several years was tested negative for Parvovirus H-1 specific antibodies, ruling out active infection and pointing to a very low level of contagiousness of H-1PV in humans (Jörg R. Schlehofer, German Cancer Research Center (DKFZ), personal communication).

The virus batch that was produced under GMP conditions for use in this clinical trial was tested extensively in animals to obtain toxicology data. Toxicology testing included intravenous and intracerebral administrations. In the repeat dose toxicology study multiple intravenous injections over a 28 day period at 3 different dose levels (low, medium, high) were employed. The only safety issue diagnosed by histology but not by clinical or laboratory abnormalities was a minimal bile duct proliferation observed in some animals of the highest and in one animal of the middle dose group after repeated applications. No other relevant pathological findings that could be attributed to H-1PV were observed, confirming that even multiple injections of high virus titers are not associated with severe side effect. Thus, injection of H-1PV according to this trial protocol can be assumed to possesses only a low risk of side effects in patients with a life threatening disease.

Due to the lack of relevant pathogenicity of H-1PV in humans, viral shedding by injected patients with subsequent symptomatic infections of unrelated subjects is extremely unlikely. In addition, patients will be isolated after virus treatment until no virus shedding can be demonstrated by real-time PCR or virus specific antibodies are present. This in combination with specific hygiene precautions further minimizes the risk for the environment and for hospital staff or other subjects.

### Statistical analysis

#### Analysis variables

The primary criterion for dose modification is the relative frequency of a dose-limiting event (DLE) at a given dose level. Toxicity of a dose is defined as the probability of this dose being associated with a DLE in a patient as indicated in the trial protocol. A dose is defined as tolerable if the probability of toxicity is lower than 33 per cent. The maximum tolerated dose is defined as the dose level with the highest posterior probability of having a toxicity not higher than 33 per cent.

#### Statistical methods

The posterior probability for toxicity will be calculated in a logistic model, where the toxicities are parametrized as follows: *p_ij_*: toxicity for group i, level j, $\text{log}\left(p_{i1}/\left(1-p_{i1}\right)\right)=a_{i}-\beta_{i;}\text{log}\left(p_{i2}/\left(1-p_{i2}\right)\right)=a_{i}\text{log}\left(p_{i3}/\left(1-p_{i3}\right)\right)=a_{i}+\beta_{i};$ with the following prior distribution for the intercept and slope parameters:

$$\left(\alpha_{1},\beta_{1},\alpha_{2},\beta_{2}\right)\sim N\left(\left(\begin{array}{c} 0\\ 0\\ 0\\ 0 \end{array}\right),\left(\begin{array}{cccc} 4 & -.2 & 1.6 & 0\\ -.2 & 4 & 0 & 1.6\\ 1.6 & 0 & 4 & -.2\\ 0 & 1.6 & -.2 & 4 \end{array}\right)\right)$$

Whenever a subject enters the study, the dose level will be elicited by calculating the posterior probability for each dose level to be tolerable. The highest dose level that is tolerable will be allocated.

The following measurements will be subject to descriptive statistical methods: vital signs, serum levels and hematology data, H-1 PV specific antibodies, virus shedding. Progression Free Survival and Overall Survival will be listed per group and subject, showing observation time and event status.

#### Dose escalation scheme

**Figure 2:**
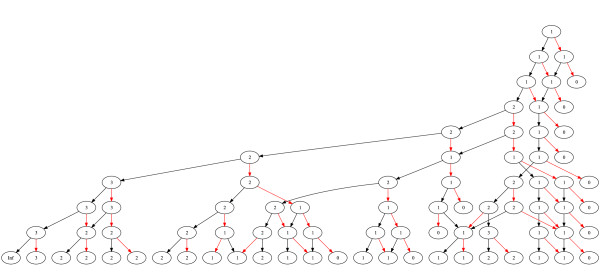
**Dose escalation scheme of ParvOryx01 for the 1^st ^study group**. The numbers (1, 2, or 3) depict the dose level for the next patient. Red arrows denote a dose limiting event in this patient and black arrows a patient tested without an event. Trial stops at the bottom line or whenever a dose level of zero is reached.

As long as no DLE is observed, a classic three-at-one design will be followed [22]. After the first DLE is observed one more patient will be recruited at the same dose before the dose will be determined as the maximum tolerated dose following the model outlined above. The highest dose with a median probability of tolerability being at least 33 per cent will be applied as the current MTD to the subsequent patient, following the Continual Reassessment Method [23]. If the MTD is ever to be calculated as being "dose 0", the trial ends prematurely at any time with the result that no safe dose could be established (Figure 2). Nine consecutive patients will be allocated to Group 1 before nine more patients will be allocated to Group 2. As the metaparameters α and β are assumed to be correlated between groups, results from group 1 affect the escalation scheme in group 2.

## Discussion

The application of replication competent oncolytic viruses is a promising approach to target dismal malignancies such as glioblastoma multiforme. The ParvOryx trial is the first in man application of oncolytic Parvovirus H-1 produced to GMP standard. To our knowledge this trial is the first clinical use of a replicating oncolytic virus in Germany. The trial design combines major efforts to assure safety with the chance to gain important information about the biological activity of the virus from specimens. Even though toxicological testing of the study medication did not reveal any safety issues after intracerebral or intravenous injection, the risk of side effects or adverse events in patients has to be minimized. To meet this demand the first 3 patients in both treatment groups will receive a low virus dose and even within each dose group patients can only be included after an extended observation period of 28 days between patient 1 and 2 and 18 days between patient 2 and 3. This concept should be capable of detecting major safety issues before a subsequent individual gets under treatment. However, long-term adverse events cannot be ruled out, but due to the aggressive nature of the disease the remaining degree of uncertainty seems justified. In this trial only patients with resectable recurrent GBMs are included. For this patient cohort the prognosis is very poor and there is no established standard of care. Tumor resection is considered beneficial irrespective of subsequent therapies. Therefore, the ParvOryx trial offers to patients the standard level of care by tumor removal in combination with the chance of oncolytic activity of the IMP. The ParvOryx trial is unique by not only including an intratumoral treatment arm, which is standard for trials with oncolytic viruses in GBM patients, but also an intravenous arm. Due to the favourable biological properties of H-1PV this trial protocol is able to reflect the possible advantage of a combined oncolytic virus treatment with a high local dose and the chance to infect remote tumor cells through the blood stream. Through the intravenous arm the ParvOryx trial will also provide information that should facilitate future clinical trials on non-CNS malignancies in which intravenous treatment is a possible route of administration.

Due to the use of an infectious albeit non-pathogenic agent specific hygienic measures have to be taken. In consequence, entering the trial will result in a temporary reduced quality of life through the need for the patient to be kept in isolation until viremia can be ruled out or seroconversion has occurred. The trial protocol takes this constraint into account by daily testing of specific probes thereby keeping the duration of isolation to a minimum. The environmental hazard of the treatment is considered to be low as H-1PV is a naturally occurring and apathogenic virus that seems to be non-contagious for humans unless directly injected. By strictly adhering to hygienic precautions the conduct of the trial should not expose others including medical staff to a relevant health risk.

The dose-finding scheme uses information from treatment group 1 when eliciting the MTD in treatment group 2. Furthermore, the final appreciation of tolerability in group 1 will retroactively use results from group 2. The rationale for this statistic approach is the assumption that at least 0.1 per cent of the viral load applied intratumorally and/or intracerebrally will enter the peripheral blood stream and the ratio between the lowest and the highest dose is 1:1000.

Finally, it is highly appreciated that by entering the trial subjects help to assess and further develop this therapeutic concept.

## Competing interests

KG holds patents related to H-1PV. JHu declares no competing interests. JR holds patents related to H-1PV. JRS holds patents related to H-1PV. MD receives salary from Oryx GmbH & Co KG. OK receives salary from Oryx GmbH & Co KG. MVKD is advisor to Oryx GmbH & Co KG. BH receives salary from Oryx GmbH & Co KG. JHa declares no competing interests.

## Authors' contributions

KG made major contributions to the study design and drafted the manuscript. JHu was responsible for the statistical concept and critically revised the manuscript. JR supervised the implementation of the virological aspects into the trial design and critically revised the manuscript. JRS contributed to the planning and implementation of virological and hygenic requirements and proofread the manuscript. BL was involved in the implementation of trial-specific requirements for trial medication and virological aspects and reviewed the manuscript. MD was involved in planning of data collection and implementation of accompanying research and critically reviewed the publication. OK planned and supervised regulatory affairs and revised the manuscript. BH coordinated the planning of the trial for the sponsor and reviewed the manuscript. MVKD participated in the study design, implemented immunological requirements and reviewed the manuscript. JHa coordinated all aspects of the trial, edited the trial documents, interacted with regulatory bodies and drafted the manuscript. All authors finally approved this publication.

### Funding

The ParvOryx01 trial is funded by Oryx GmbH & Co KG, Baldham, Germany.

## Pre-publication history

The pre-publication history for this paper can be accessed here:

http://www.biomedcentral.com/1471-2407/12/99/prepub
